# Complement Receptor C5aR1 Inhibition Reduces Pyroptosis in hDPP4-Transgenic Mice Infected with MERS-CoV

**DOI:** 10.3390/v11010039

**Published:** 2019-01-09

**Authors:** Yuting Jiang, Junfeng Li, Yue Teng, Hong Sun, Guang Tian, Lei He, Pei Li, Yuehong Chen, Yan Guo, Jiangfan Li, Guangyu Zhao, Yusen Zhou, Shihui Sun

**Affiliations:** 1 State Key Laboratory of Pathogen and Biosecurity, Beijing Institute of Microbiology and Epidemiology, Beijing 100071, China; captain99@126.com (Y.J.); lijunfeng2113@126.com (J.L.); yueteng@me.com (Y.T.); tglnx05@tom.com (G.T.); helei_happy@126.com (L.H.); lwhisperer@163.com (P.L.); chenyuehong.happy@163.com (Y.C.); muhan0425@126.com (Y.G.); anatee@163.com (J.L.) guangyu0525@163.com (G.Z.); 2 Department of Basic Medical Sciences, North China University of Science and Technology, Tangshan 063210, China; xun1@hotmail.com; 3 Institute of Medical and Pharmaceutical Sciences, Zhengzhou University, Zhengzhou 450052, China

**Keywords:** MERS-CoV, inflammation, pyroptosis, complement

## Abstract

Middle East respiratory syndrome coronavirus (MERS-CoV) is a highly pathogenic virus with a crude mortality rate of ~35%. Previously, we established a human DPP4 transgenic (hDPP4-Tg) mouse model in which we studied complement overactivation-induced immunopathogenesis. Here, to better understand the pathogenesis of MERS-CoV, we studied the role of pyroptosis in THP-1 cells and hDPP4 Tg mice with MERS-CoV infection. We found that MERS-CoV infection induced pyroptosis and over-activation of complement in human macrophages. The hDPP4-Tg mice infected with MERS-CoV overexpressed caspase-1 in the spleen and showed high IL-1β levels in serum, suggesting that pyroptosis occurred after infection. However, when the C5a-C5aR1 axis was blocked by an anti-C5aR1 antibody (Ab), expression of caspase-1 and IL-1β fell. These data indicate that MERS-CoV infection induces overactivation of complement, which may contribute to pyroptosis and inflammation. Pyroptosis and inflammation were suppressed by inhibiting C5aR1. These results will further our understanding of the pathogenesis of MERS-CoV infection.

## 1. Introduction

Middle East respiratory syndrome coronavirus (MERS-CoV), the second highly pathogenic coronavirus to emerge after severe acute respiratory syndrome coronavirus (SARS-CoV), causes severe acute respiratory failure and extra-pulmonary multi-organ damage accompanied by severe systemic inflammation [1,2,3]. However, the pathogenesis of MERS-CoV still needs to be explored. Complement activation and pyroptosis are two proteolytic cascades that defend the host against dangerous pathogens. They are important parts of the innate immune system and have some similar characteristics, including pore-formation and proinflammatory characteristics.

Pyroptosis is a lytic and inflammatory mode of regulated cell death catalyzed by the caspase family [4]. Activation of caspase-1 relies on assembly of inflammasome complexes, which contain NLRP1b, NLRC4, NLRP3, and AIM2. Different inflammasomes are activated by different pathogen-associated molecular patterns (PAMPs) or danger-associated molecular patterns (DAMPs) via particular pattern recognition receptors (PRRs). The best-characterized inflammasome is the NLRP3 inflammasome, which responds to a variety of bacterial, viral, and fungal agents [5,6], DAMPs (e.g., ATP, monosodium urate crystals, and amyloid-β aggregates) [7,8], and even environmental and industrial particles such as silica and asbestos [9]. The NLRP3 inflammasome comprises the NLRP3 scaffold, the ASC (PYCARD) adaptor, and pro-caspase-1. The activated NLRP3 inflammasome promotes transformation of pro-caspase-1 to its active form, which proteolytically cleaves gasdermin D, pro-IL-1β, and pro-IL-18 to yield their bioactive forms. The N-terminal domain of cleaved gasdermin D perforates the cell membrane, resulting in osmotic lysis [10], whereas mature IL-1β and IL-18 act as proinflammatory cytokines [11].

The complement system is an ancient molecular cascade; indeed, homologs have been found in sea urchin [12] and mosquitoes [13]. Complement is activated via three pathways: the classical, lectin, and alternative pathways. During the process of activation, an enzyme named C3 convertase cleaves C3 to C3a and C3b, which are recruited to the C3 convertase to form the C5 convertase. C5 convertase catalyzes cleavage of C5 to C5a and C5b to initiate the terminal complement pathway, resulting in formation of the membrane attack complex, which has pore-forming properties. During this process, two split products, C3a and C5a (known as anaphylatoxins), promote inflammation or serve as chemoattractants by engaging their cognate receptors [14,15].

In a previous study we demonstrated that aberrant complement activation contributes to severe outcomes in hDPP4 transgenic mice infected with MERS-CoV, and that preventing over-activation of the complement system may be an effective clinical therapy for MERS [16]. Here, we examined the role of pyroptosis in the pathogenesis of MERS, along with the relationship between pyroptosis and complement. The results may help us to better understand the mechanism underlying severe outcomes after MERS-CoV infection.

## 2. Materials and Methods

### 2.1. Ethics Statement

All animal experiments were approved by the Institutional Animal Care and Use Committee (IACUC) of the Beijing Institute of Microbiology and Epidemiology (IACUC Permit No: BIME 2017-0011; Permit Date: 8 March 2017). Animal studies were carried out in strict accordance with the recommendations set out in the Guide for the Care and Use of Laboratory Animals.

### 2.2. Cells

Human monocytic cells (THP-1) were purchased from the American Type Culture Collection (Manassas, VA, USA, ATCC Number: TIB-202) and cultured in RPMI 1640 medium supplemented with 10% heat-inactivated fetal bovine serum (FBS), 100 U/mL penicillin, 100 μg/mL streptomycin sulfate, 1× Glutamax-I (l-glutamine alternate), and 0.05 mM 2-mercaptoethanol. THP-1 (differentiated) macrophages were obtained by exposing the cells to 60 nM phorbol-12-myristate-13-acetate (PMA) for 12 h, followed by culture for a further 24 h in complete growth medium without PMA.

### 2.3. Virus Infection

MERS-CoV (HCoV-EMC/2012 strain) was propagated and titrated on Vero cells in an approved biosafety level 3 laboratory. THP-1 differentiated macrophages cultured in 75-cm^2^ flasks were infected with MERS-CoV at a multiplicity of infection of 0.1. The virus was adsorbed at 37 °C for 1 h and unbound virus was washed away.

HDPP4-Tg mice (6 weeks old, female) [17] were maintained in a pathogen-free facility and housed in cages containing sterilized feed and drinking water. Following intraperitoneal anesthetization with sodium pentobarbital (5 mg/kg body weight), mice were inoculated intranasally with MERS-CoV (103.3 50% tissue culture infectious dose (TCID50)) in 20 μL Dulbecco’s modified Eagle’s medium (DMEM). Mice in the sham group received the same volume of DMEM. For the experiments of C5aR1 inhibition, mice were received an intravenous (i.v.) injection (600 μg/kg) of a monoclonal Ab (mAb) specific for mouse C5aR1 (Hycult Biotech, Uden, the Netherlands) to block the interaction of C5a to C5aR1 or an injection of phosphate-buffered saline (PBS) (sham treatment control) at the same time as MERS-CoV inoculation. All infectious experiments related to MERS-CoV were performed in an approved biosafety level 3 facility.

### 2.4. Isolation of RNA and Proteins

THP-1 differentiated macrophages were lysed in TRIzol™ Reagent (Life Technologies, Carlsbad, CA, USA) at 24 h post-infection with MERS-CoV. Total RNA and proteins were isolated according to the reagent user guide.

Mice were euthanized by overdose inhalation of carbon dioxide at different time points after infection with MERS-CoV. Lungs were harvested and total RNA was extracted and purified using an RNeasy Extraction Kit (Qiagen, Hilden, Germany).

### 2.5. Quantitative Reverse Transcription-PCR

To detect expression of inflammasomes and complement components in MERS-CoV-infected THP-1 differentiated macrophages and hDPP4-Tg mice, 2 μg of total RNA from cells or the lung of mice we used as template for first-strand cDNA synthesis. The resulting cDNA was subjected to quantitative PCR using Power SYBR^®^ Green PCR Master Mix (Life Technologies, Carlsbad, CA, USA) to determine the relative abundance of inflammasome and complement components. The forward and reverse primers used for each component are listed in the Table 1. The relative amount of each gene was obtained by normalization against an endogenous control gene (GAPDH) and calculated using the comparative 2^−ΔΔCT^ method. Sham-infected monocytes and sham-infected hDPP4-Tg mice were used as respective calibrators. An identical amplification reaction comprising (i) polymerase activation and DNA denaturation at 95 °C for 10 min, (ii) 40 cycles each of the denaturation at 95 °C for 10 s, and (iii) an annealing/extension step at 60 °C for 30 s, was used for each gene analyzed.

### 2.6. Western Blot Analysis

Proteins isolated from THP-1 monocytic cells and THP-1 differentiated macrophages were electrophoresed in a 12% SDS-PAGE gel and transferred to a PVDF membrane (GE Healthcare, Dassel, Germany). PVDF membranes were then blocked for 1 h at room temperature in 5% non-fat milk. Membranes were incubated overnight at 4 °C with primary antibodies, followed by secondary antibodies for 1 h at room temperature. The primary antibodies used for analysis were: mouse anti-caspase 1 (1:200; Santa Cruz Biotechnology, Cat: sc-392736, Santa Cruz, CA, USA), mouse anti-IL-1β (1:200; Santa Cruz Biotechnology, Cat: sc-32294), rabbit anti-cleaved IL-1β (1:2000; Cell Signaling, Cat: #83186, Danvers, MA, USA), rabbit anti-MERS-CoV Nucleocapsid Protein (1:2000; Sino Biological Inc., Cat: 40068-RP02, Beijing, China), and rabbit anti-β-actin (1:2000; Cell Signaling, Cat: #4970). The secondary antibody was anti-rabbit secondary (1:5000; TransGen, Cat: HS101-01, Beijing, China) or anti-mouse secondary (1:5000; TransGen Cat: HS201-01). Blots were developed using a Pierce ECL Western Blotting Substrate (Thermo Scientific, Waltham, MA, USA) and protein bands were detected using an Amersham Imager 600 (GE Healthcare, Chicago, IL, USA).

### 2.7. Analysis of Inflammatory Cytokines

Cytokines in mouse serum were measured using a Milliplex Mouse Cytokine/Chemokine Magnetic Panel Kit (Merck Millipore, Burlington, MA, USA). A panel of inflammatory cytokines (IL-1β, IL-6, TNF-α, and IFN-γ) was detected according to the manufacturer’s protocol.

### 2.8. Immunohistochemistry (IHC)

Sections of paraffin-embedded spleen and lung tissues (4 μm thick) were prepared and stained to detect antigen expression. Briefly, retrieved sections were incubated overnight at 4 °C with the following antibodies: mouse anti-caspase-1 mAb (AdipoGen, San Diego, CA, USA), polyclonal rabbit anti-CD68 (Abcam, Cambridge, MA, USA), and polyclonal anti-IFN-γRα (Santa Cruz Biotechnology). Biotinylated immunoglobulin G was then added, followed by an avidin–biotin–peroxidase conjugate (Beijing Zhongshan Biotechnology Co., Ltd., Beijing, China). Immunoreactivity was detected using 3,3′ diamino benzidine (DAB). Slides were counter-stained with hematoxylin.

### 2.9. Statistical Analysis

Statistical analyses were performed using GraphPad Prism software, version 5.01 (GraphPad Software, San Diego, CA, USA). Student’s *t* test was used to compare two groups with respect to relative expression of mRNA and cytokine levels in serum. *p* values < 0.05 were considered significant.

## 3. Results

### 3.1. MERS-CoV Infection Induced Pyroptosis in THP-1 Macrophages

**Figure 1:**
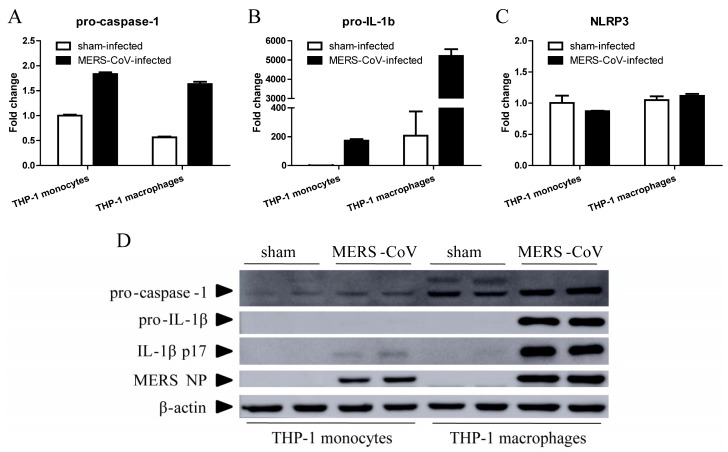
MERS-CoV infection induces pyroptosis in THP-1 macrophages. THP-1 monocytes and macrophages were infected with MERS-CoV for 24 h. Total RNA and protein was then extracted from the cells using TRIzol Reagent. (**A**–**C**) Total RNA was used for RT-qPCR to detect transcription of pro-caspase-1, pro-IL-1β, and NLRP3. Data are expressed as means ± SEM (*n* = 2 per group). (**D**) Samples of total protein were subjected to Western blotting to detect pro-caspase-1, pro-IL-1β, activated IL-1β, and MERS NP.

Unlike abortive infection of SARS-CoV in human macrophages, MERS-CoV can establish a productive infection in macrophages and induce production of proinflammatory cytokines and chemokines [18]. Many RNA viruses, such as EV71, H1N1, H7N9 influenza A virus, and Zika virus, can infect macrophages and trigger IL-1β secretion via the NLRP3 inflammasome [19,20,21,22]. To evaluate the response of macrophages to MERS-CoV infection, we inoculated THP-1 monocytic cells and THP-1 differentiated macrophages with MERS-CoV or RPMI 1640 medium (sham-infection). We then examined expression of NLRP3, pro-caspase-1, and pro-IL-1β 24 h later by RT-qPCR. As shown in Figure 1, MERS-CoV infection induced relatively higher expression of pro-caspase-1 (Figure 1A) and pro-IL-1β (Figure 1B), but not NLRP3 (Figure 1C), in both THP-1 monocytes and macrophages. Expression of pro-IL-1β in monocytes increased by 170-fold, whereas that in macrophages increased by 26-fold (on average).

We verified expression of caspase-1, IL-1β, and MERS nucleocapsid protein (NP) by Western blotting (Figure 1D). MERS-CoV-infected THP-1 macrophages expressed higher levels of pro-caspase-1, pro-IL-1β, and activated IL-1β (p17) than sham-infected THP-1 macrophages or MERS-CoV-infected THP-1 monocytes. MERS NP was detected in both MERS-CoV-infected THP-1 monocytes and macrophages. These results indicate that MERS-CoV infection induces high levels of proinflammatory IL-1β secretion and THP-1 macrophage pyroptosis.

### 3.2. Pyroptosis in Mice Infected with MERS-CoV

**Figure 2:**
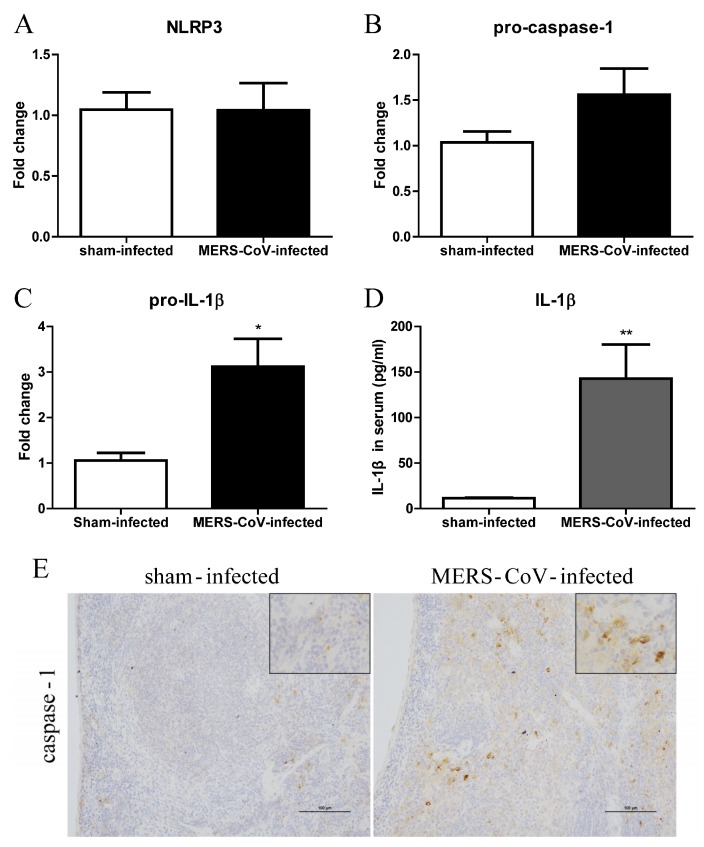
MERS-CoV infection induces pyroptosis in hDPP4-transgenic mice. (**A**–**C**) Transcription of NLRP3, pro-caspase-1, and pro-IL-1β in lung tissue at Day 3 post-MERS-CoV infection (*n* = 5–6 per group). (**D**) Concentration of IL-1β in serum at Day 3 post-MERS-CoV infection. Data are expressed as means ± SEM (*n* = 5–6 per group). * *p* < 0.05, ** *p* < 0.01 (Student’s *t* test with Welch’s correction). (**E**) Representative images of immunohistochemical staining of caspase-1 in lung tissue on Day 7 post-challenge of sham-infected and MERS-CoV-infected mice (scale bars = 100 μm).

To determine whether MERS-CoV infection induces pyroptosis in mice, we used RT-qPCR to detect mRNA encoding NLRP3, pro-caspase-1, and pro-IL-1β in lung tissue from hDPP4 transgenic mice at Day 3 post-MERS-CoV infection. Although there was no significant difference in expression of NLRP3 and pro-caspase-1 between the sham-infected and MERS-CoV-infected groups (Figure 2A,B), expression of pro-IL-1β mRNA was significantly higher after MERS-CoV infection (Figure 2C). In addition, we measured the concentration of IL-1β in serum. The results showed that MERS-CoV infection induced production of IL-1β (Figure 2D). Furthermore, we examined expression of caspase-1 in the lung and spleen at Day 7 post-MERS-CoV infection by IHC. In line with the mRNA results, there was no significant difference in expression of caspase-1 in the lung of sham-infected and MERS-CoV-infected mice. However, the spleens of mice infected with MERS-CoV showed higher expression of caspase-1 than those of mice in the sham group (Figure 2E). The results indicated that MERS-CoV infection could induce pyroptosis in mice.

### 3.3. Inflammatory Responses in Mice Infected with MERS-CoV

**Figure 3:**
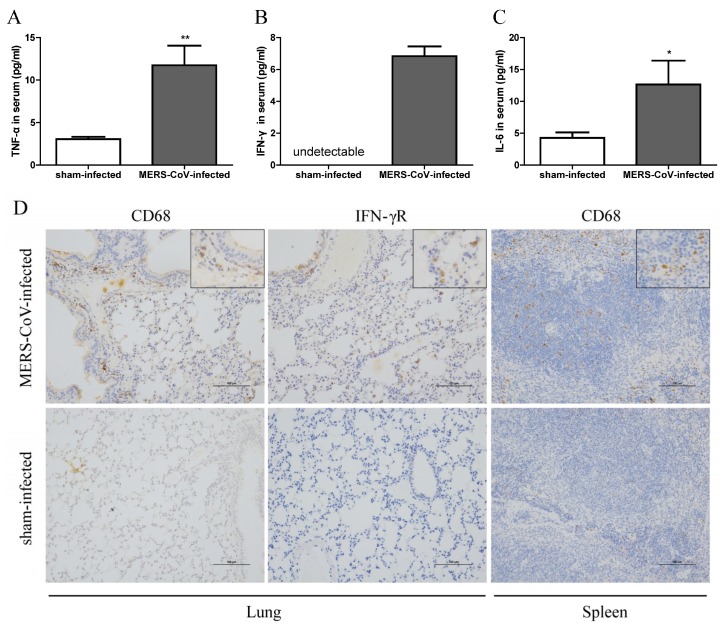
MERS-CoV infection induces systemic inflammation in hDPP4-transgenic mice. (**A**–**C**) Concentration of TNF-α, IFN-γ, and IL-6 in serum at Day 3 post-MERS-CoV infection. Data are expressed as means ± SEM (*n* = 5–6 per group). * *p* < 0.05, ** *p* < 0.01 (Student’s *t* test with Welch’s correction). (**D**) Macrophage infiltration and expression of IFN-γ receptor were assessed by immunohistochemical staining of lung and spleen at 7 days post-challenge (scale bars = 100 μm).

IL-1β plays an important role in mediating autoinflammatory diseases and in generating inflammatory responses to infection [23]. Therefore, to assess the inflammatory responses in mice, we measured TNF-α, IFN-γ, and IL-6 in serum at Day 3 post-MERS-CoV infection. As shown in Figure 3A–C, serum from mice in the MERS-CoV-infected group contained more TNF-α, IFN-γ, and IL-6 than that from sham-infected mice. IHC examination of CD68 and IFN-γ receptor expression also suggested greater macrophage infiltration and activation in the lung and spleen of mice at 7 days post-MERS-CoV infection (Figure 3D). These results indicate that MERS-CoV infection causes systemic inflammation, as reported in clinical MERS patients and MERS-CoV infected animal models [17,24].

### 3.4. MERS-CoV Infection Alters Expression of Complement in THP-1 Monocytes and Macrophages

**Figure 4:**
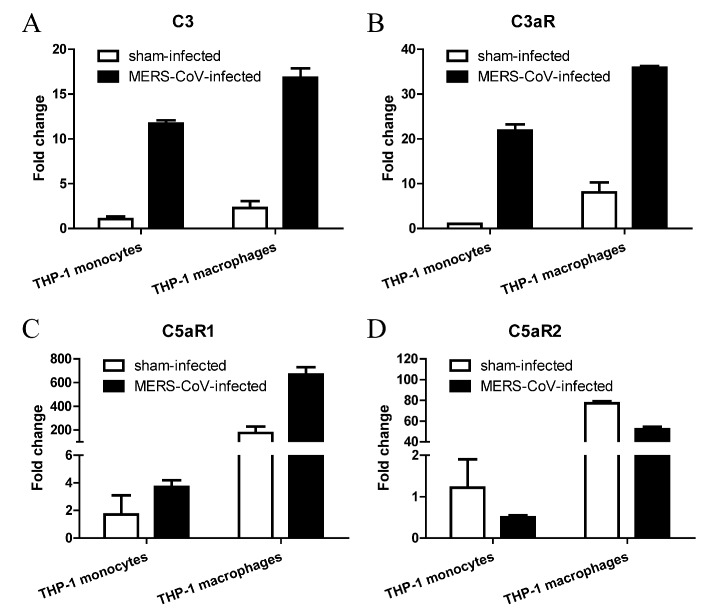
MERS-CoV infection alters complement expression in monocytes and macrophages. (**A**–**D**) Transcription of C3, C3aR, C5aR1, and C5aR2 in THP-1 monocytes and macrophages at 24 h post-MERS-CoV infection. Data are expressed as means ± SEM (*n* = 2 per group).

The complement system links activation of Toll-like receptors to transcription of IL-1β mRNA [25,26]. It has been studied that intracellular C3 is converted to biologically active C3a and C3b by the protease cathepsin L [27], and C3a activates NLRP3 and triggers IL-1β production in human monocytes by regulating efflux of ATP [28]. In addition, C5a is believed to induce a proinflammatory or anti-inflammatory response when ligated to C5aR1 or C5aR2 respectively [29,30,31]. Thus, we used RT-qPCR to examine expression of complement components and their receptors C3aR, C5aR1, and C5aR2. As shown in Figure 4, C3 and C3aR expression by both THP-1 monocytes and macrophages was highly upregulated (by 5–20-fold) after MERS-CoV infection. C5aR1 was upregulated, whereas C5aR2 was downregulated, after MERS-CoV infection.

### 3.5. Inhibiting C5aR1 Reduces Pyroptosis in Mice Infected with MERS-CoV

**Figure 5:**
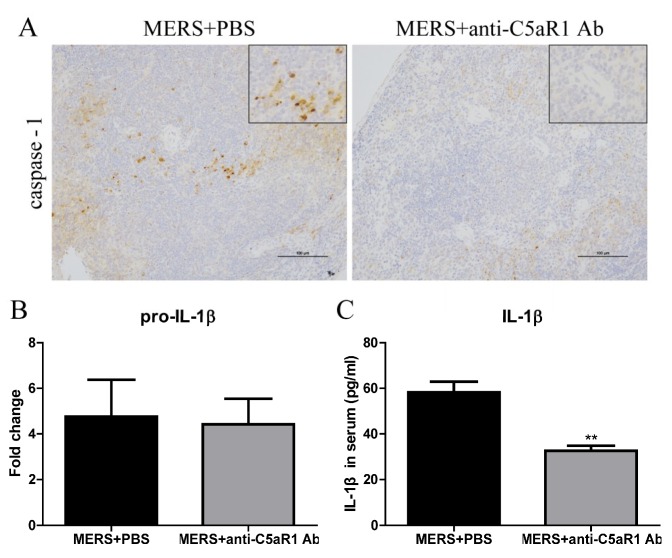
Blocking the C5a-C5aR1 axis reduces pyroptosis in hDPP4-transgenic mice. (**A**) Representative images of immunohistochemical staining of caspase-1 in lungs from PBS-treated and anti-C5aR1 Ab-treated groups on Day 7 post-challenge (scale bars = 100 μm). (**B**) Transcription of pro-IL-1β in lung tissues from PBS-treated and anti-C5aR1 Ab-treated groups at Day 7 post-infection (*n* = 4–5 per group). (**C**) Concentration of IL-1β in serum at Day 1 post-MERS-CoV infection. Data are expressed as means ± SEM (*n* = 4–5 per group). ** *p* < 0.01 (Student’s *t* test with Welch’s correction).

Our previous study demonstrated that MERS-CoV infection results in dysregulated host immune responses and severe tissue damage [17] and inhibiting C5aR1 alleviates MERS-CoV infection-induced tissue damage by regulating host immune responses [16]. Here, we used an anti-C5aR1 Ab to block the C5a-C5aR1 axis. The antibody was administered at the same time as MERS-CoV infection. We then measured expression of caspase-1 in the spleen and IL-1β in the lung and serum. Compared with the PBS-treated group, mice receiving the anti-C5aR1 Ab expressed less caspase-1 in the spleen at Day 7 post-MERS-CoV infection (Figure 5A). Although there was no significant difference between the two groups with respect to pro-IL-1β mRNA expression (Figure 5B), serum levels of IL-1β were lower in the anti-C5aR1 Ab-treated group than in the PBS-treated group at Day 1 (Figure 5C) and Day 3 [16] post-MERS-CoV infection. These results suggest that complement inhibition decreased the expression of pyroptosis indicators, IL-1β and caspase-1, in mice infected with MERS-CoV.

### 3.6. Inhibiting C5aR1 Reduces Inflammation in Mice Infected with MERS-CoV

**Figure 6:**
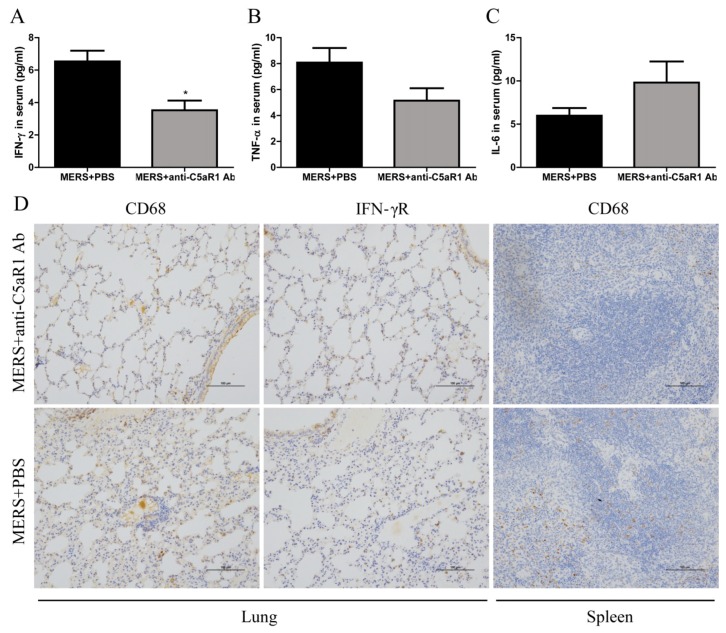
Blockade of the C5a-C5aR1 axis reduces pyroptosis in hDPP4-transgenic mice. (**A**–**C**) Concentration of IFN-γ, TNF-α, and IL-6 in serum at Day 1 post-MERS-CoV infection. Data are expressed as means ± SEM (*n* = 4–5 per group). * *p* < 0.05 (Student’s *t* test with Welch’s correction). (**D**) Infiltration of the lungs and spleen by macrophages and expression of IFN-γ receptor were assessed by immunohistochemical staining 7 days after challenge (scale bars = 100 μm).

At 1 day after MERS-CoV infection, we measured proinflammatory cytokines (IFN-γ, TNF-α, and IL-6) in serum. IFN-γ levels in mice treated with the anti-C5aR1 Ab were much lower than those in the PBS-treated group (Figure 6A). To further evaluate the effect of complement inhibition on the local inflammation at later time after MERS-CoV infection, we examined expression of CD68 and IFN-γ receptor in lung and spleen at 7 days post-MERS-CoV infection. IHC revealed that macrophage infiltration and activation were lower in the anti-C5aR1 Ab-treated group (Figure 6D). Taken together, these results suggest that inhibiting complement dampens the over-activated inflammatory response in mice infected with MERS-CoV.

## 4. Discussion

Macrophages play important roles in host defense by clearing dead cells, ingesting and destroying microbes, and presenting antigens to T lymphocytes. In addition, macrophages produce the full array of complement components [32] and PRRs, which are closely associated with inflammasome activation and pyroptosis. The accumulated studies indicate that macrophages play an important role in the pathogenesis of SARS and MERS [18,33]. Macrophages infected with MERS-CoV secrete proinflammatory cytokines and chemokines [18]. Widespread distribution of these macrophages throughout many organs is one of the reasons underlying multi-organ damage and systemic inflammation after virus infection. Pyroptosis or inflammasome activation plays an important role in virus-mediated pathogenesis. For example, abortive HIV-1 infection in quiescent lymphoid CD4 T cells leads to CD4 T cell pyroptosis independent of the NLRP3 inflammasome [34]. EV71 3D and ZIKV NS5 activate the NLRP3 inflammasome by interacting with NACHT and the LRR domain of NLRP3 [19,22]. The PB1-F2 protein of avian influenza A virus H1N1 or H7N9 induces inflammation by activating the NLRP3 inflammasome [35,36], and deficiency of NLRP3 or caspase-1 protects mice against H7N9 infection-associated morbidity and mortality [21]. Here, we demonstrate that the dysregulated macrophage-mediated immune responses after MERS-CoV infection may contribute to a severe outcome.

Several studies demonstrate relationships between complement and inflammasomes. For example, C3-/- mice display reduced inflammasome activation in an intracerebral hemorrhage (ICH) model [37]. Engagement of C5a and C5aR1 on CD4+ T cells generates reactive oxygen species, which are a classical DAMP, thereby triggering inflammasome assembly [38]. In monocytes, C3a alters metabolic programming and increases ATP efflux, leading to NLRP3 activation and IL-1β generation via the receptor P2 × 7 [28]. Thus, the complement system is considered to be an essential regulatory component of the cellular alarm system that controls inflammasome activation [39]. Here, we inhibit MERS-CoV infection-induced inflammation and pyroptosis by blocking the C5a-C5aR1 axis with an anti-C5aR1 antibody.

In our study, we first demonstrated that virus infection leads to pyroptosis by measuring activation of caspase-1 and IL-1β in human macrophages infected with MERS-CoV (Figure 1). Next, we found that MERS-CoV infection induced transcription of pro-IL-1β in the lung and expression of caspase-1 in the spleen. Activated caspase-1 could be released into the extracellular space and delivered to the lung via exosomes [40] or the systemic circulation; it then cleaves pro-IL-1β into its bio-activated form, which is secreted into the serum (Figure 2D). We cannot exclude the possibility that pyroptosis occurs in the spleen because we did not detect expression of pro-IL-1β. Meanwhile, examination of CD68 and IFN-γ receptor expression revealed macrophage infiltration and activation in the lungs and spleen (Figure 3D). Thus, pyroptosis could occur in both organs.

In our previous study, we showed that inhibiting C5aR1 increased splenic cell regeneration and decreased splenic cell apoptosis, thereby alleviating MERS-CoV infection-induced tissue damage [16]. The spleen happens to be a large reservoir for myeloid lineage cells such as macrophages, dendritic cells and CD4+ T cells in which pyroptosis mainly occurred [4,34,41]. Here, we studied and demonstrated that inhibiting C5aR1 suppressed caspase-1 activation (Figure 5A) and macrophage infiltration and activation in the spleen (Figure 6D), and thereby reduced the secretion of IL-1β into the serum (Figure 5C) and the systemic inflammation (Figure 6). However, in the lung tissue, there was no significant difference of pro-IL-1β transcription between the Ab and sham-treated groups (Figure 5B), which may due to the limited macrophages, the main cell type in which pyroptosis occurred, when compared to that in spleen. 

**Figure 7:**
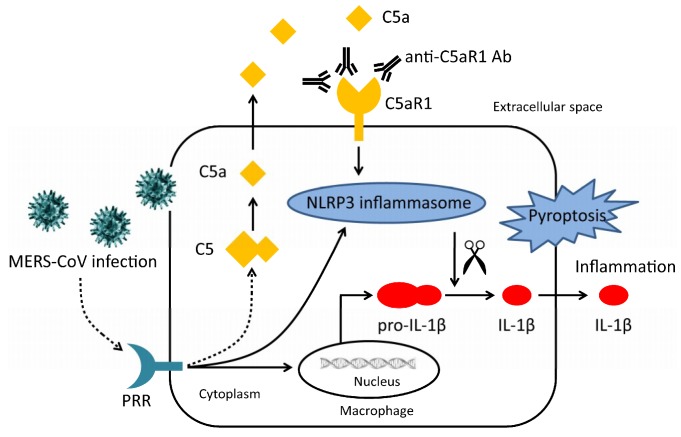
Diagram illustrating the relationship between complement and pyroptosis during MERS-CoV infection. MERS-CoV infection induces activation of complement and transcription of pro-IL-1β. Activated complement component C5a is released into the extracellular space where it interacts with C5aR1, which triggers assembly of the NLRP3 inflammasome. Activated caspase-1 cleaves pro-IL-1β into its active form thereby triggering pyroptosis. An anti-C5aR1 antibody prevents interaction between C5a and C5aR1, thereby ameliorating pyroptosis.

Although many studies have focused on the link between complement and pyroptosis, the pathways that link them remain unclear. Here, our results showed that MERS-CoV infection induces pro-IL-1β transcription, and complement activation, which leads to pyroptosis in macrophages. Induction of pyroptosis was related with complement activation and may be promoted by ligation of C5a and C5aR1, which was confirmed by the blockade of anti-C5aR1 antibody (Figure 7).

In summary, these data indicate that MERS-CoV infection induces overactivation of complement, which may contribute to pyroptosis and inflammation. Pyroptosis and inflammation were suppressed by inhibiting C5aR1. These research will further our understanding of the pathogenesis of MERS-CoV infection.

## Figures and Tables

**Table 1:** Primers used to amplify inflammasome and complement components.

Primer	Species	Gene	Orientation	Sequence (5′-3′)
1	Human	NLRP3	F	ATTCGGAGATTGTGGTTGGG
			R	AGGGCGTTGTCACTCAGGTC
2		pro-caspase-1	F	CTCAGGCTCAGAAGGGAATGTC
			R	TGTGCGGCTTGACTTGTCC
3		pro-IL-1b	F	GCTCGCCAGTGAAATGATGG
			R	CAGAGGGCAGAGGTCCAGG
4		C3	F	CACTATGATCCTTGAGATCTGTACCA
			R	GGAGCAAAGCCAGTCATCA
5		C3aR	F	GACATCCAGGTGCTGAAGCC
			R	ACTGGGGGCTCATTCCATG
6		C5aR1	F	GCTGACCATACCCTCCTTCCT
			R	CCGTTTGTCGTGGCTGTAGTC
7		C5aR2	F	TGCTGTTTGTCTCTGCCCATC
			R	GTCAGCAGGATGATGGAGGG
8	Mouse	pro-caspase-1	F	AATGAAGTTGCTGCTGGAGGA
			R	CAGAAGTCTTGTGCTCTGGGC
9		pro-IL-1b	F	TGGACCTTCCAGGATGAGGACA
			R	GTTCATCTCGGAGCCTGTAGTG
